# Jump Rope Training Improves Muscular Strength and Cardiovascular Fitness in University Students: A Controlled Educational Intervention

**DOI:** 10.3390/sports13090307

**Published:** 2025-09-05

**Authors:** Sabău Anca Maria, Ordean Mircea Nicolae, Mancini Nicola, Alexandra Szara Szekely, Simon Sorin, Ianc Dorina, Carlos Hervás-Gómez, Popovici Cornelia, Grosu Emilia Florina, Grosu Vlad Teodor

**Affiliations:** 1 Department of Physical Education, Sport and Physiotherapy, University of Oradea, 410087 Oradea, Romania; asabau@uoradea.ro (S.A.M.); szekelyszara15@yahoo.com (A.S.S.); dianc@uoradea.ro (I.D.); 2 Department of Physical Education and Sports, “December 1, 1918” University, Strada Gabriel Bethlen 5, 510009 Alba Iulia, Romania; sorin.simon@uab.ro; 3 Department of Education and Sport Sciences, Pegaso Telematic University, 80143 Naples, Italy; 4 Department of Teaching and Educational Organization, University of Sevilla, 41013 Sevilla, Spain; hervas@us.es; 5 Department of Medical Education, Medicine Faculty, Physical Education, “Iuliu Hațieganu” University of Medicine and Pharmacy in Cluj-Napoca, Victor Babeș Street 8, 400347 Cluj-Napoca, Romania; popovici.cornelia@umfcluj.ro; 6 Faculty of Physical Education and Sport, “Babes-Bolyai” University, Pandurilor Street 7, 400394 Cluj-Napoca, Romania; emilia.grosu@ubbcluj.ro; 7 Faculty of Industrial Engineering, Robotics, and Production Management, Technical University of Cluj-Napoca, Muncii Street 103–105, 400461 Cluj-Napoca, Romania; vlad.grosu@mdm.utcluj.ro

**Keywords:** jump rope, muscular strength, Ruffier test, physical education, cardiovascular capacity, university students

## Abstract

This study aimed to evaluate the effects of jump rope training on cardiovascular capacity, assessed with the Ruffier test, and muscular strength, measured using isometric dynamometry (BioFET Mustec, Almere, The Netherlands), within a university physical education program. A total of 52 undergraduate students from non-specialist faculties at the University of Oradea were randomly assigned to either an experimental group (EG) or a control group (CG). Over eight weeks, the EG performed a ten-minute jump rope training session once per week in combination with cardiovascular exercises, while the CG participated only in cardiovascular exercises. Statistical analyses revealed a significant group effect on cardiovascular response (Ruffier Index: *p* = 0.019; Cohen’s d = −0.271) and a substantial increase in right lower limb strength (*p* = 0.003; d = 1.026) in the EG compared to the CG. Furthermore, improvements were observed in upper limb strength (left arm: *p* = 0.010; d = 0.922) and left lower limb strength (*p* = 0.027; d = 0.779). These findings suggest that incorporating jump rope training into university physical education classes may represent an effective and low-cost strategy to enhance both cardiovascular efficiency and muscular strength in young adults. Given its simplicity and affordability, jump rope training appears feasible for implementation in schools and universities, even where resources are limited. Future research should investigate its long-term effects across different populations.

## 1. Introduction

The promotion of cardiovascular and muscular health is a primary goal in physical education. Among low-cost tools, jump rope training stands out for its effectiveness in improving endurance, coordination, and strength. Despite its documented benefits, jump rope is rarely included in university-level physical education programs [1]. It represents a functional and engaging educational strategy capable of stimulating multiple domains of fitness. Rope skipping is a complex motor skill that requires the coordinated rotation of a rope with the arms and repeated bilateral jumps. This activity combines plyometric effort with rhythm and coordination, thereby highlighting its potential to develop motor control and physical conditioning [2]. In this study, the jump rope protocol involved 10 minutes of training per session, once a week for 8 weeks, integrated into a 30-min physical education class. The activity progressed from low-intensity alternate skipping to more complex and continuous jump variations, ensuring a gradual adaptation process.

Jump rope is a versatile workout that combines fitness, recreation, and competition. It has been described by European and American medical experts as one of the most complete health exercises [3], and it is also recommended by the American Heart Association and the British Osteoporosis Society to improve physical activity at all stages of life [4]. In physical education, various pedagogical approaches are adopted, ranging from traditional skill-drill methods to game-based learning, circuit training, and cooperative activities, each targeting specific physical and cognitive outcomes. Within this framework, experimental educational studies test structured programs under controlled conditions to evaluate their effectiveness in enhancing students’ fitness and engagement. The present study adopts this approach, assessing the integration of jump rope training into regular PE classes.

Jumping rope requires coordination between the upper and lower limbs, and different movement patterns (e.g., weighted rope exercises) can be used to flexibly control the elements of movement, support neuromuscular regulation, and promote adaptive responses in the central nervous system [5]. Furthermore, children and teenagers can increase their physical fitness levels in a fun and competitive way by jumping rope, which is often more engaging than traditional physical education classes [6].

With a participation rate of 11.2%, jumping rope is the second most popular activity among youngsters in China, after running [7]. Recent years have also seen an increase in the use of jump rope in physical education classes and during recess [8,9]. This is consistent with [10], which states that compared with other aerobic workouts, jumping rope enhances students’ physical fitness.

Cardiovascular endurance is defined as the combined ability of the pulmonary system to promote gas exchange, the cardiovascular system to transport oxygen, and the muscular system to utilize it during sustained physical effort [11]. According to current recommendations from the World Health Organization and the American College of Sports Medicine, adults and young adults should engage in at least 150 min per weeks of moderate-intensity aerobic activity, or 75 min of vigorous activity, ideally distributed across multiple days. In educational settings, aerobic exercise should be prescribed with progressive overload, ensuring adequate warm-up, maintaining intensity between 60–85% of age-predicted maximum heart rate, and incorporating variety to sustain motivation. These principles guided the integration of jump rope training into the present experimental program.

Regular aerobic activities such as rope jumping have been associated with improved oxygen extraction, enhanced circulatory efficiency, and musculoskeletal strengthening [12]. Recent biomechanical studies have shown that jump rope exercises engage both the lower and upper limbs, enhancing whole-body coordination and performance [13]. Moreover, systematic reviews have confirmed the positive effects of jump rope training on health- and sport-related physical fitness, particularly in improving muscular strength and cardiovascular function [14]. Jump rope is also used in sports training as a practical tool to develop conditioning, balance, and motor abilities [15]. Its simplicity and versatility make it a suitable exercise even in educational contexts [16].

The purpose of this study was to evaluate the effects of jump rope training on cardiovascular capacity and muscular strength within a university physical education program. Based on previous evidence, we aimed to determine whether the integration of a structured jump rope program into PE classes could enhance upper and lower limb muscular strength and cardiovascular capacity in young adults. Consistent with standard experimental design, three research questions were tested using a null versus alternative hypothesis model:

1. Jump rope training will significantly improve upper limb strength.
2. Jump rope training will significantly improve lower limb strength.
3. Jump rope training will significantly improve cardiovascular capacity.

The null hypothesis in each case assumes no difference between the experimental and control groups.

## 2. Materials and Methods

### 2.1. Participants

A total of 52 university students, evenly split by sex (13 males and 13 females in each group), aged between 18 and 20 years, took part in the study (EG: *n* = 26; CG: *n* = 26). Participants were recruited through non-probabilistic convenience sampling from undergraduate students regularly attending PE classes at the University of Oradea. Once eligibility was confirmed, participants were randomly assigned to EG or CG using simple randomization, manually generated from a numbered list of eligible students.

Eligibility criteria were as follows: enrollment in a non-specialist undergraduate program, regular attendance at physical education classes, and absence of cardiovascular and/or musculoskeletal disorders. Exclusion criteria included current injuries, missing more than 25% of the planned sessions, or the use of medication likely to affect physical performance. All enrolled participants (*n* = 52) completed the study, and no dropouts were recorded. Table 1 presents the baseline anthropometric characteristics of the participants.

**Figure 1:**
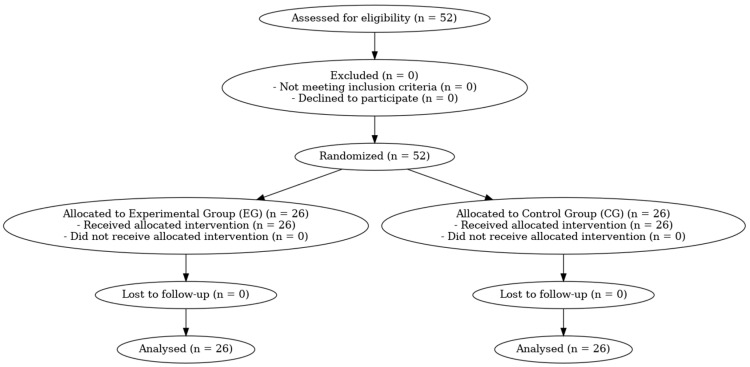
CONSORT-style flow diagram of participant enrollment, allocation, follow-up, and analysis. A total of 52 students were assessed for eligibility; none were excluded; 52 were randomized (EG = 26; CG = 26); all received the allocated intervention; no loss to follow-up occurred; and all participants were included in the final analyses.

A CONSORT-style flow diagram illustrating enrollment, allocation, follow-up, and analysis is shown in Figure 1.

### 2.2. Experimental Design and Intervention Protocol

The study adopted a two-arm, parallel-group, pretest–posttest design, including an experimental group (EG) and a control group (CG). The intervention lasted eight weeks (March–May), with one session per week, conducted during the second semester of the 2023–2024 academic year as part of regular physical education classes at the University of Oradea. The EG followed a structured 30-min routine consisting of 20 min of bodyweight cardiovascular exercises (e.g., jumping jacks, skipping, and running in place), followed by 10 min of continuous, self-paced jump rope training. Both groups participated in the same 30-min weekly sessions; however, only the EG performed the jump rope component. To ensure uniformity, all activities were standardized and supervised by the same teacher.

Protocol design guidelines. The intervention was developed in accordance with established recommendations for aerobic exercise in educational settings, emphasizing moderate intensity, progressive overload, safety, and variety to sustain engagement. The weekly progression and work–rest structure were based on public health and PE guidelines, as well as jump rope-specific evidence. The program progressed from low-complexity alternate skipping to more continuous and complex sequences, with brief and structured recovery intervals [3,9,11,13,14,15,17].

In Table 2, we list all the activities completed during the eight weeks, in detail.

The study was conducted in accordance with the ethical principles of the Declaration of Helsinki (2013) and was approved by the Institutional Review Board—Ethics Committee on Scientific Research Conduct and Ethics Representatives of the Faculty of Geography, Tourism and Sport, University of Oradea, Romania (protocol code No. 571/25 April 2024). All participants were adults, voluntarily agreed to participate, and provided informed consent prior to their involvement in the intervention.

### 2.3. Instruments

To assess the effects of the intervention, two validated instruments were used.

The Ruffier Test, a submaximal cardiovascular endurance assessment, was administered according to established procedures [17,18]. Participants performed 30 consecutive squats in 45 s. Heart rate was recorded at rest before the exercise (*P*_0_), immediately after completing the squats (*P*_1_), and after 1 min of recovery (*P*_2_). The Ruffier Index was calculated using the following formula:

***Index* = [(*P*_0_ + *P*_1_ + *P*_2_) − 200]/10**,

where lower scores indicate better cardiovascular efficiency. The interpretation of the results was as follows: <0 = very good; 0–5 = good; 5–10 = satisfactory; 10–15 = moderate; 15–20 = poor; >20 = very poor.

The isometric strength test was performed using a BioFET digital dynamometer (Mustec, Almere, The Netherlands), a device designed to measure maximal isometric strength in the upper and lower limbs. The BioFET has demonstrated high validity and excellent internal consistency for isometric muscle strength assessment (ICC > 0.90) [19]. The device records peak isometric force in kilograms-force (kgf) with a resolution of 0.1 kgf, as well as the duration of effort (seconds).

For the upper limb assessment, participants were seated with the elbow flexed at 90°, forearm in a neutral position, and shoulder adducted, performing elbow flexion against the resistance applied by the dynamometer handle positioned on the forearm. For the lower limb assessment, participants were seated with the knee flexed at 90°, ankle secured to the device strap, and instructed to extend the leg maximally against the strap’s resistance. Each contraction lasted 5 s, with two repetitions per limb and a 1-min rest interval between trials. The dynamometer and chair were calibrated before each testing session according to the manufacturer’s specifications.

The device is widely used in both rehabilitation and sports settings for the development and monitoring of strength training programs. This test is particularly suited to young adult populations because it is quick to administer, requires minimal equipment, and provides a reliable measure of muscular strength in educational settings.

### 2.4. Statistical Analysis

All analyses were conducted using SPSS software (version 25.0; IBM Corp., Chicago, IL, USA). Data were preliminarily screened to confirm the absence of outliers and to verify distributional assumptions. Quantitative variables were described using the mean, standard deviation, and 95% confidence intervals. Additionally, for descriptive purposes, the variation Δ (post-test [T1] − baseline [T0]) was calculated for each group.

The normality of the distributions was assessed using the Shapiro–Wilk test (appropriate for small samples, *n* < 50), and homogeneity of variances was verified with Levene’s test. For within-group comparisons (baseline [T0] vs. post-test [T1]), paired-sample t-tests were performed separately for the experimental group (EG) and the control group (CG). Between-group comparisons (EG vs. CG at post-test [T1]) were conducted using independent-sample t-tests.

For variables showing significant changes, a two-way repeated-measures analysis of variance (2 × 2 ANOVA) was subsequently performed, with one between-subjects factor (group: EG vs. CG) and one within-subjects factor (time: T0 vs. T1). This analysis allowed the assessment of main effects for time and group, as well as the group × time interaction, which reflects the specific effectiveness of the intervention.

The level of statistical significance was set at *p* < 0.05. For all statistically significant results, effect sizes (Cohen’s d) were also calculated and interpreted according to international guidelines: d = 0.2 (small), d = 0.5 (medium), and d ≥ 0.8 (large).

Finally, the sample size was estimated a priori using G*Power software (version 3.1), assuming α = 0.05, power = 0.80, and f = 0.25 (medium effect size). This calculation indicated that a minimum of 12 participants per group was sufficient for the planned 2 × 2 repeated-measures ANOVA.

## 3. Results

The analyses conducted using 2 × 2 ANOVA (group × time) revealed the following effects:

Ruffier Test: A significant main effect of group was found (F = 5.666, *p* = 0.019), indicating an overall difference between the experimental group (EG) and the control group (CG). No significant effects of time (*p* = 0.367) or group × time interaction (*p* = 0.705) were observed. However, within-group analysis for the EG showed a clinically relevant improvement in the index, with a reduction from 7.80 ± 2.82 to 7.07 ± 2.56 and a Cohen’s d = −0.271 (95% CI [−0.49, −0.05], small effect). Although the effect size was small, this reduction reflects a meaningful enhancement in cardiovascular recovery capacity, particularly relevant in educational contexts where training time is limited.

Upper Limb Strength:

Left upper limb: A significant effect of time (F = 6.915, *p* = 0.010) and a group × time interaction (F = 4.633, *p* = 0.034) were found, indicating a specific improvement in the EG. The mean value increased from 8.44 ± 1.85 to 10.17 ± 1.89, with a Cohen’s d = 0.922 (95% CI [0.47, 1.35], large effect).

Right upper limb: A significant effect of time was observed (F = 7.766, *p* = 0.006), but no effects of group or interaction. In the EG, the increase from 8.53 ± 1.90 to 10.17 ± 1.92 corresponds to a Cohen’s d = 0.858 (95% CI [0.42, 1.29], large effect).

Lower Limb Strength:

Left lower limb: Significant effects were found for both group (F = 5.169, *p* = 0.025) and time (F = 5.066, *p* = 0.027); the group × time interaction approached statistical significance (*p* = 0.057), without reaching the conventional threshold. Although not statistically significant, this finding suggests a favorable trend regarding the intervention’s effectiveness. In the EG, the increase from 9.06 ± 1.51 to 10.54 ± 2.22 resulted in a Cohen’s d = 0.779 (95% CI [0.34, 1.20], moderate-to-large effect). This gain represents a substantial improvement in lower limb strength, with functional relevance for daily activities and sports performance in young adults.

Right lower limb: Significant effects were found for group (F = 4.006, *p* = 0.048), time (F = 9.437, *p* = 0.003), and group × time interaction (F = 5.031, *p* = 0.027). The improvement from 8.83 ± 1.44 to 10.67 ± 2.10 in the EG corresponds to a Cohen’s d = 1.026 (95% CI [0.57, 1.46], very large effect). Such a large effect indicates that the intervention yielded highly meaningful gains in maximal lower limb strength, surpassing typical improvements expected from standard physical education programs.

Overall, the findings demonstrate that the regular inclusion of jump rope in the university physical education program produced statistically significant improvements, with effect sizes ranging from moderate to very large for upper and lower limb strength, and a small but meaningful improvement in cardiovascular capacity.

As shown in Table 3, the mean values in the isometric strength tests remained relatively stable in the control group, while a clear increase was observed in the experimental group between T0 and T1. Internal variability, as indicated by the baseline standard deviation range across measured variables (SD: 1.44–3.23 for performance outcomes; 1.44–8.55 including anthropometric measures), remained low and consistent, supporting the homogeneity of the two groups at T0.

Specifically, in the EG, the average gain in lower limb strength exceeded 1.5 kg, while the Ruffier Index showed an average decrease of approximately 0.7 points, consistent with enhanced cardiovascular efficiency.

Table 4 presents the results of the two-way analysis of variance (2 × 2 ANOVA) for each variable analyzed. For the Ruffier Index, a significant main effect of group was observed (*p* = 0.019), indicating an overall difference between the experimental group (EG) and the control group (CG). For left upper limb strength, both the main effect of time (*p* = 0.010) and the group × time interaction (*p* = 0.034) were significant, highlighting a specific improvement in the EG. Right upper limb strength showed a significant effect of time (*p* = 0.006) but no significant group or interaction effects.

For the lower limbs, significant main effects were observed for both group and time (all *p* < 0.05). In addition, right lower limb strength exhibited a significant group × time interaction (*p* = 0.027), while the interaction for the left lower limb approached significance (*p* = 0.057), suggesting a favorable trend in the EG.

Table 5 summarizes the magnitude of change within the experimental group (EG) through Cohen’s d values for each variable. The largest effect was observed for right lower limb strength (d = 1.026), followed by left upper limb strength (d = 0.922) and right upper limb strength (d = 0.858), all of which are classified as large effects. Improvement in left lower limb strength showed a moderate-to-large effect (d = 0.779). Although the Ruffier Index yielded a small effect size (d = −0.271), the negative value reflects an enhancement in cardiovascular efficiency. Collectively, these results confirm that the jump rope intervention produced meaningful improvements in both muscular strength and cardiovascular adaptation within the experimental group.

**Figure 2:**
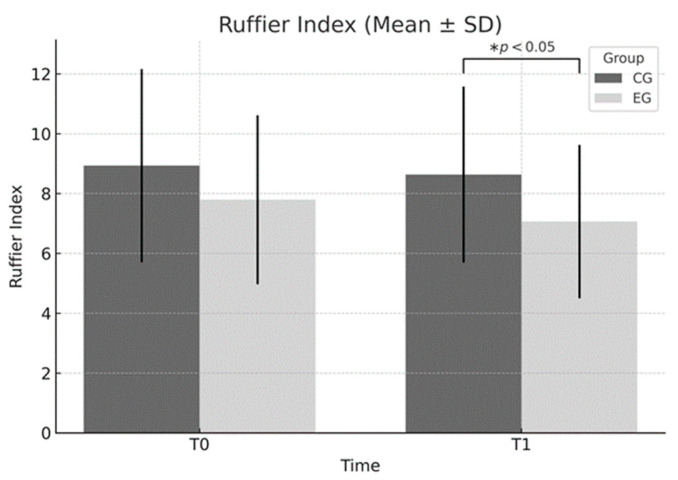
Ruffier Index (mean ± SD) at baseline (T0) and post-intervention (T1) in the experimental (EG) and control (CG) groups. A significant main effect of group was observed (F = 5.666, *p* = 0.019), indicating better overall cardiovascular efficiency in the EG. No significant time × group interaction was found. * *p* < 0.05 between groups at T1.

As illustrated in Figure 2, the Ruffier Index decreased slightly in the experimental group (EG) from T0 to T1, while remaining stable in the control group (CG). Although no significant group × time interaction was found, a statistically significant main effect of group (*p* = 0.019) indicated overall better cardiovascular efficiency in the EG.

**Figure 3:**
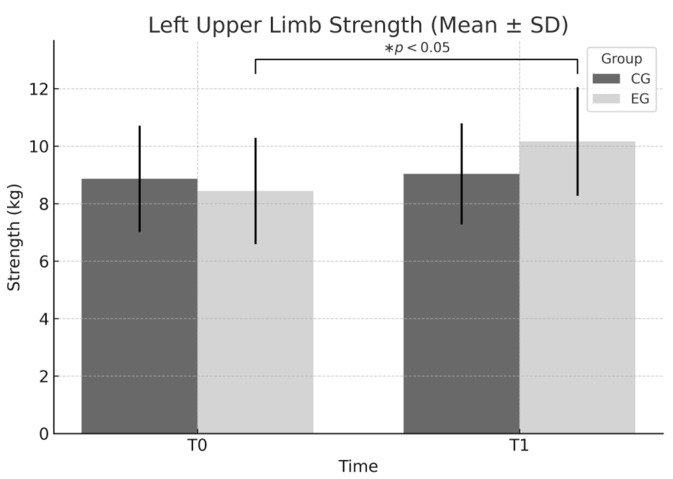
Left upper limb strength (mean ± SD) at T0 and T1 for EG and CG. Significant main effect of time (F = 6.915, *p* = 0.010) and significant time × group interaction (F = 4.633, *p* = 0.034) indicate specific improvement in the EG. * *p* < 0.05 within-group improvement from T0 to T1 in the EG.

Figure 3 shows a notable increase in left upper limb strength in the EG following the intervention, whereas the CG maintained similar values across time points. This trend is statistically confirmed by both a significant time effect (*p* = 0.010) and a group × time interaction (*p* = 0.034).

**Figure 4:**
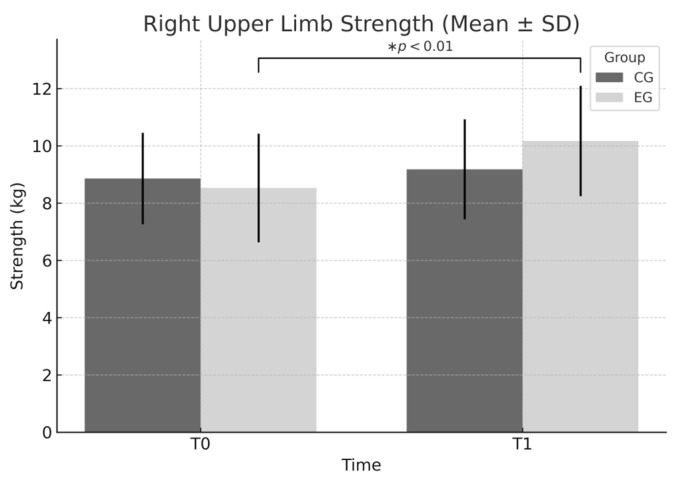
Right upper limb strength (mean ± SD) at T0 and T1 in EG and CG. Significant main effect of time (F = 7.766, *p* = 0.006) observed, with both groups improving over time; however, the EG showed a larger mean increase. No significant group or interaction effects were found. * *p* < 0.01 within-group improvement in the EG.

According to Figure 4, both groups improved in right upper limb strength over time; however, the EG exhibited a greater mean increase. This finding is supported by a statistically significant main effect of time (*p* = 0.006), although neither the group effect nor the interaction effect reached significance.

**Figure 5:**
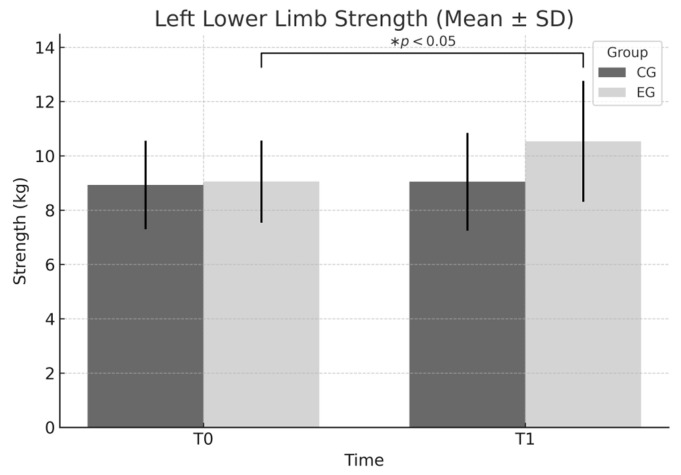
Left lower limb strength (mean ± SD) at T0 and T1 for EG and CG. Significant main effects were found for group (F = 5.169, *p* = 0.025) and time (F = 5.066, *p* = 0.027), while the group × time interaction approached significance (*p* = 0.057). * *p* < 0.05 within-group improvement from T0 to T1 in the EG.

In Figure 5, left lower limb strength appears to improve more substantially in the EG compared to the CG. This is consistent with the significant main effects for both time (*p* = 0.027) and group (*p* = 0.025), while the interaction effect approached significance (*p* = 0.057).

**Figure 6:**
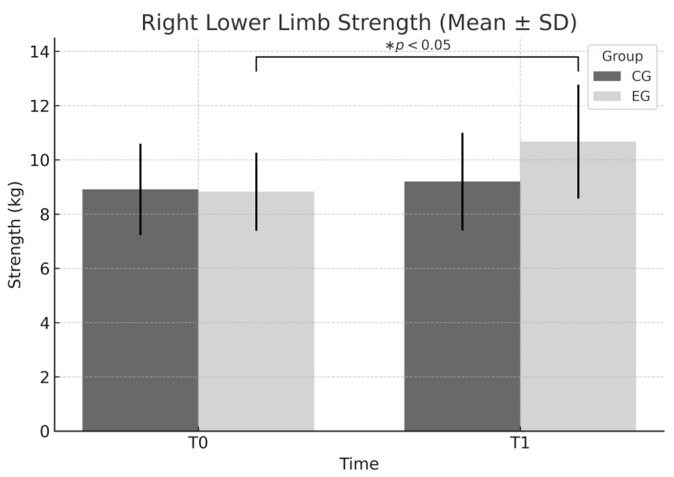
Right lower limb strength (mean ± SD) at T0 and T1 in EG and CG. Significant main effects for group (F = 4.006, *p* = 0.048), time (F = 9.437, *p* = 0.003), and group × time interaction (F = 5.031, *p* = 0.027) indicate a markedly greater improvement in the EG. * *p* < 0.05 within-group improvement from T0 to T1 in the EG.

Figure 6 displays the largest gain in strength, particularly in the right lower limb of the EG. This result is supported by statistically significant effects for group (*p* = 0.048), time (*p* = 0.003), and their interaction (*p* = 0.027), indicating the strongest response to the training protocol.

**Figure 7:**
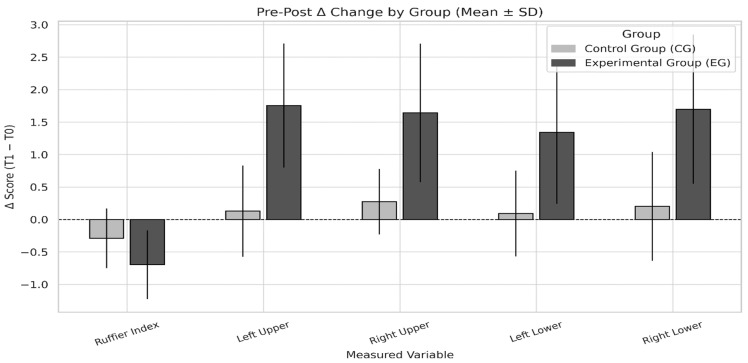
Δ-change (T1 − T0) in each measured variable for EG and CG (mean ± SD). Variables include Ruffier Index, left/right upper limb strength, and left/right lower limb strength. Positive deltas represent increases in strength; negative delta in the Ruffier Index indicates improved cardiovascular efficiency. The EG showed larger positive deltas for all strength measures and a greater reduction in the Ruffier Index compared to the CG.

Visual inspection of Figure 7 suggests greater overall improvements in the EG for all tested variables. Positive deltas were evident in all strength tests, while the Ruffier Index showed a beneficial negative delta.

These results reinforce the efficacy of rope jumping in enhancing both muscular and cardiovascular outcomes.

## 4. Discussion

This study investigated the effects of incorporating a structured jump rope module into a university physical education program on muscular strength and cardiovascular efficiency in non-specialist undergraduates. The findings indicate that regular participation in jump rope activities led to notable improvements in lower-limb isometric strength and a positive adaptation in cardiovascular response, supporting its potential as a simple and low-cost enhancement to standard PE classes.

The most pronounced gains were found in lower-limb strength, especially in the right limb, where the intervention produced marked improvements. The left lower limb also improved substantially, though the pattern of change was slightly less marked. These results likely reflect neuromuscular adaptations to the plyometric, cyclic, and rhythmic demands of rope skipping, which emphasizes repetitive stretch–shortening cycles and sustained muscular engagement. In contrast, upper-limb strength improvements were smaller. A meaningful change was observed for the left upper limb, while the right upper limb showed only a general increase over time without a clear interaction effect. This asymmetry may be attributed to the functional role of the arms in rope skipping, which primarily contribute through cyclical rope rotation and coordination rather than sustained force production.

The cardiovascular data showed a beneficial reduction in the Ruffier Index for the experimental group, indicating improved recovery and efficiency. While the magnitude of change was modest compared to strength outcomes, the improvement is noteworthy in an educational setting, where time and resources for high-intensity endurance work are often limited. Although the effect size for the Ruffier Index was small (d = −0.271), such a reduction likely reflects meaningful early gains in cardiovascular health and recovery capacity. In the context of young adults, even modest gains can serve as an important foundation for longer-term cardiovascular adaptations, particularly when integrated into regular physical education programs.

Similar benefits have been observed in previous studies. For instance, ref. [16] reported enhanced cardiovascular endurance after a 7-week rope-skipping program in adolescents, and ref. [20] found increased VO_2_max in university students following jump rope training. It should be emphasized, however, that VO_2_max was not assessed in the present study. These findings are consistent with broader evidence identifying jump rope as a cost-effective and accessible method for enhancing cardiorespiratory fitness, coordination, and neuromuscular control [20]. Prior research also highlights its suitability in educational environments due to its simplicity and positive influence on student engagement and adherence [7]. Evidence from [20] also underlines its role in improving leg strength, speed, and aerobic capacity, while [21] highlights how structured, varied, and play-based programs can stimulate motor coordination more effectively than traditional PE formats. Intervention parameters, such as frequency, duration, and progression, are important in determining outcomes. Subgroup analyses suggest that protocols lasting 8–12 weeks, performed twice weekly for ≥40 min, are optimal for developing physical fitness in pediatric populations (e.g., preadolescents aged 10–12) [22]. These parameters, however, may not directly translate to university students.

For children aged 9–10, acute jump rope activities—particularly those incorporating HIIT or MICT—have been shown to improve working memory, executive control, and orienting network efficiency [23], and additional studies have demonstrated positive effects on physical fitness in female pupils of the same age [24]. While this pediatric evidence underscores the versatility of jump rope training, our findings in young adults indicate that even a brief, once-weekly 10-min protocol embedded in PE classes can produce meaningful strength gains and modest improvements in cardiovascular recovery. Therefore, age-specific dosing, frequency, and progression should be considered when adapting school-based protocols to university contexts.

It is also important to note that much of the existing literature on jump rope training involves pediatric populations. While these studies provide valuable context, the present findings contribute specifically to the evidence base on young adults enrolled in university programs.

Some studies have documented cases of tibial periostitis related to excessive rope skipping, underscoring the importance of progressive overload and appropriate volume control [25]. Variability in sample sizes, program design, and participant demographics partly explains the mixed findings in the literature. For example, ref. [15] reported no positive effects on certain morphological indicators, while [20] observed increases in height among boys. Chen Y. and Du F. reported contrasting results in agility performance among girls [26,27]. Rope skipping has also been successfully applied in adolescents with mild intellectual disabilities, where gradual progression improved exercise tolerance, cardiovascular health, and overall physical fitness [28,29]. However, some research found no effect on BMI in these populations, despite significant gains in cardiovascular endurance, muscular strength, and endurance capacity [30]. Improvements in static and dynamic balance have also been documented in male students with intellectual disabilities [31].

The week-to-week progression adopted in this study—from easy alternate skipping to continuous sequences with varied jumps—offers a practical, scalable model for university PE classes. The activity’s low cost, minimal space requirements, and adaptability to different skill levels make it a particularly attractive option for promoting active lifestyles in educational settings. Nevertheless, several limitations should be acknowledged, including the relatively short duration of the intervention, the absence of long-term follow-up, and the specific sample of non-specialist undergraduates, which may limit generalizability. Moreover, although both male and female students participated, analyses were not stratified by sex, and therefore, potential gender-based differences in response to jump rope training could not be assessed.

Future research should explore higher training frequencies, longer program durations, and direct comparisons with other aerobic–plyometric modalities. In addition, the inclusion of behavioral outcomes such as enjoyment and adherence, along with wearable-based monitoring, could help to define optimal implementation strategies across diverse populations.

### Limitations and Future Directions

This study presents some limitations that should be acknowledged. The relatively short duration of the intervention (only eight weeks with one session per week) may have restricted the magnitude of the observed effects, and the absence of a follow-up evaluation limited the ability to assess long-term retention of adaptations. Another potential limitation is the uncontrolled individual variability in baseline fitness levels and extracurricular physical activity, which might have influenced the outcomes. Moreover, although valid, the performance metrics employed were somewhat limited, as they did not include objective assessments such as jump height or VO_2_ max, which could have provided a more comprehensive understanding of the participants’ adaptations. To address these limitations, future research could extend both the duration and frequency of the intervention, for example by implementing two to three sessions per week over a twelve-week period, to capture more robust and sustained training effects. Comparative studies are also encouraged to evaluate the effectiveness of jump rope training against other plyometric or aerobic modalities, such as high-intensity interval training (HIIT). Furthermore, the inclusion of psychological outcomes—such as perceived exertion, motivation, or overall well-being—would provide a more holistic perspective on the benefits of such interventions. Finally, the use of wearable devices could enhance the accuracy of monitoring by capturing both internal load parameters (e.g., heart rate variability) and subjective feedback (e.g., ratings of perceived exertion and enjoyment), thereby offering a richer understanding of participants’ responses

## 5. Conclusions

This study demonstrated that integrating rope skipping into university physical education classes produced significant improvements in lower-limb isometric strength and a reduction in the Ruffier Index, indicating enhanced cardiovascular efficiency. These findings support the use of brief, low-cost jump rope interventions as an effective strategy to improve muscular strength and cardiovascular response in non-specialist undergraduate students. For successful large-scale adoption and sustainable implementation in educational settings, institutional support, structured program delivery, and consistent monitoring will be essential.

## Figures and Tables

**Table 1:** Anthropometric characteristics of participants at baseline (T0) (mean ± standard deviation), divided by group: experimental group (EG) and control group (CG).

Characteristics	Experimental Group (EG)	Control Group (CG)
Body height (cm)	156.17 ± 8.09	156.50 ± 6.19
Body mass (kg)	50.50 ± 8.55	49.75 ± 7.48
BMI (kg/m^2^)	20.61 ± 2.67	20.23 ± 2.18

Values are presented as mean ± SD; BMI: body mass index.

**Table 2:** Activities completed during the eight weeks, in detail.

Week	Total Duration (min)	Activities—Aerobic Gymnastics (First 20 min)	Other Activities (Only EG— 10 min Jump Rope)	Observations
1	30	Step-and-run variations Slow activation of the major bodily parts Zone work, exercises in high and low positions, and dynamic stretching	Alternate skipping rope with forward rotation at a leisurely pace—breaks as required (brief, self-regulated by participants)	Overview and acquaintance with the movements
2	30	Step and run variations Gradual engagement of the main body segments Exercises from high and low positions, working on zones Active stretching	Alternate skipping rope with forward rotation at a moderate pace—breaks as required	Emphasis on coordination and posture
3	30	Variations of steps and running in place with arm movements Gradual engagement of the main body segments Exercises from high and low positions, working on zones Combined stretching	Skipping rope alternately, at a moderate pace, front and backward Brief pauses when necessary	Developing mobility and strength
4	30	Variants of walking and running in place and on the move with arm movements Gradual engagement of the main body segments Exercises from high and low positions, working on zones Stretching	Alternate jump ropes, forward 30 s breaks between sets	Developing mobility and strength
5	30	Basic aerobic steps, such as step touch, knee lift and grapevine Gradual engagement of the main body segments High and low position exercises, working on zones Active stretching	Alternate skipping rope, rotating the rope forward and backward, in rhythmic series Short breaks between series	Gradual increase in intensity
6	30	Basic aerobic gymnastics steps, such as step touch, knee lift and grapevine, combined with arm movements Progressive activation of body segments: head—shoulders—trunk—limbs Exercises performed from high and low positions	Continuous jump rope with various jump variants and rhythmic shifts Brief rest periods in between sets	Increasing intensity
7	30	Step variations that involve both upper and lower limb synchronization and movement Engaging in effort the upper and lower segments	Continuous jump rope, with different jump variations, 5 sets × 1.30 min—30 s break between sets	
8	30	Step variations that combine movement with upper and lower limb synchronization General warm-up by successively involving body segments Exercises performed from high and low positions	Continuous jump rope, with different jump variations, 4 sets × 2 min—1 min break between sets	

**Table 3:** Isometric strength and cardiovascular measures (mean ± SD) at T0 and T1 for EG and CG.

Group	Time	Ruffier_Mean SD	L_Upper_Strength Mean SD	R_Upper_Strength Mean SD	L_Lower_Strength Mean SD	R_Lower_Strength Mean SD
CG	T0	8.93 ± 3.23	8.87 ± 1.85	8.86 ± 1.6	8.93 ± 1.63	8.91 ± 1.68
CG	T1	8.63 ± 2.94	9.04 ± 1.76	9.18 ± 1.74	9.05 ± 1.8	9.2 ± 1.8
EG	T0	7.8 ± 2.82	8.44 ± 1.85	8.53 ± 1.9	9.06 ± 1.51	8.83 ± 1.44
EG	T1	7.07 ± 2.56	10.17 ± 1.89	10.17 ± 1.92	10.54 ± 2.22	10.67 ± 2.1

**Table 4:** Results of the two-way analysis of variance (2 × 2 ANOVA) for the main effects of group, time, and their interaction for each analyzed variable. F values, *p*-values, and partial eta-squared (η^2^p) are reported.

Variable	F Group	P Group	η^2^p Group	F Time	P Time	η^2^p Time	F Interaction	*p* Interaction	η^2^p Interaction
Ruffier Index	5.666	0.019	0.102	0.820	0.367	0.016	0.144	0.705	0.003
Left Upper Limb Strength	0.956	0.331	0.019	6.915	0.010	0.121	4.633	0.034	0.085
Right Upper Limb Strength	0.875	0.352	0.017	7.766	0.006	0.134	3.500	0.064	0.065
Left Lower Limb Strength	5.169	0.025	0.094	5.066	0.027	0.092	3.693	0.057	0.069
Right Lower Limb Strength	4.006	0.048	0.074	9.437	0.003	0.159	5.031	0.027	0.091

**Table 5:** Cohen’s d values for pre-post comparisons in the experimental group (EG) for each variable.

Variable	Cohen’s d	Effect Size Interpretation
Ruffier Index	−0.271	Small (beneficial)
Left Upper Limb Strength	0.922	Large
Right Upper Limb Strength	0.858	Large
Left Lower Limb Strength	0.779	Moderate to large
Right Lower Limb Strength	1.026	Very large

Note. Effect sizes are interpreted according to Cohen’s guidelines: small = 0.2, moderate = 0.5, large ≥ 0.8. Negative value for Ruffier indicates improvement (lower score = better cardiovascular adaptation).

## Data Availability

The data presented in this study are available on request from the corresponding author.
